# Purse-string versus linear closure of the skin wound following stoma reversal: A meta-analysis with RCT and systematic review

**DOI:** 10.1097/MD.0000000000039477

**Published:** 2024-08-30

**Authors:** Jinlong Luo, Dan Liu, Junmei Wu, Huaiwu Jiang, Jin Chen, Hua Yang, Lie Yang

**Affiliations:** a Department of Colorectal Anal Surgery, Zigong Fourth People’s Hospital, Zigong, Sichuan Province, China; b Department of Gastrointestinal Oncology, The First Affiliated Hospital of Hainan Medical University, Haikou, Hainan Province, China; c Department of General Surgery, Sichuan Mianyang 404 Hospital, Mianyang, Sichuan Province, China; d Division of Gastrointestinal Surgery, Department of General Surgery, West China Hospital, Sichuan Province, China.

**Keywords:** linear closure, meta-analysis, purse-string closure, surgical site infection

## Abstract

**Background:**

Linear closure (LC) following stoma reversal is associated with a high risk of surgical site infection (SSI). Purse-string closure and LC were both positive for the closure of the skin wound following stoma reversal, and it was not yet possible to distinguish which one was more beneficial to the patient’s prognosis.

**Methods:**

We conducted a search in Embase, PubMed, Web of Science, and Cochrane Library and conducted a randomized controlled experiment from the inception of each database to July 2024. Among them, the SSI within 30 days, operation time, hospitalization time, incisional hernia, and wound healing time were all outcome indicators.

**Results:**

Eleven studies were included in this meta-analysis (506 patients in the purse-string closure group and 489 patients in the LC group). The pooled data showed that the SSI rate was significantly lower in the purse-string closure group than in the LC group (odds ratio, 0.15; 95% confidence interval, 0.09–0.24; *P* < .00001; I^2^ = 0%). The differences in operative time, hospitalization time, incision hernia, and wound healing time were not statistically significant.

**Conclusion:**

Overall, purse-string closure had a significantly lower SSI rate following stoma reversal than LC.

## 1. Introduction

Temporary stoma is generally performed in colorectal surgery to decrease the risk of anastomotic leakage and reoperation.^[1]^ Surgical site infection (SSI) is one of the most common complications associated with surgery.^[2]^ Linear closure (LC) is used in stoma reversal surgery, and SSI rates have been reported to be as high as 41%.^[3]^ The increased incidence of incisional hernia, delayed wound healing, decreased patient satisfaction, prolonged hospitalization time, and increased hospitalization expenses may be caused by SSI.^[4]^

In 1997, Banerjee^[5]^ reported a surgical method called purse-string closure (PSC) that decreased scarring, which helped reduce the incidence of SSI. The fascial closure procedure leaves a gap of approximately 5 mm after the skin is stitched. By draining the residual hematoma or exudates, the incision healed secondarily, thereby reducing the risk of SSI. We reviewed 11 randomized controlled trials (RCTs) to compare the SSI rate, operation time, hospitalization time, wound healing time, and incidence of incisional hernias.

## 2. Materials and methods

### 2.1. Search strategy

A comprehensive search of all published RCTs was performed to compare the surgical outcomes of PSC versus LC following stoma reversal surgery.

We searched Embase, PubMed, China National Knowledge Infrastructure, Web of Science, Technology Journal Database, and Wanfang Database from the inception of each database to July 2024. The search terms included “purse string,” “linear closure,” and “colostomy/ileostomy reversal.” A gray literature search of the US National Institutes of Health trial register (clinicaltrials.gov) was also performed. The references included in the articles were checked and analyzed further. In any case, a third reviewer discussed or negotiated to resolve any differences between the 2 authors. The current research program was registered in the Prospective Registry of International Systematic Evaluation (ID: CRD42022311080). Our study followed the Preferred Items for Reporting of Systematic Reviews and Meta-Analyses^[6]^ and Meta-Analysis of Observational Studies in Epidemiology guidelines.^[7]^

### 2.2. Selection criteria and explanations

RCTs comparing PSC and LC for wound closure following stoma reversal must focus on one of the outcomes, including SSI, incisional hernia, operative time, hospital stay, and wound healing time.

According to the US Centers for Disease Control and Prevention guidelines, SSI is a superficial or deep incisional infection within 30 days after surgery.^[8]^

### 2.3. Data extraction and quality assessment

Two reviewers independently extracted the outcomes including the first author, country, year of publication, stoma type, antibiotic use, follow-up, and outcome data (SSI, incision hernia, operative time, hospital stay, and wound healing time). In the event of disagreement, the final decision was made through discussion or consultation with a third reviewer.

Two authors independently assessed the risk of bias.^[9]^ When there was disagreement between the 2 authors, a third author was consulted. The following 7 domains were estimated: random sequence generation, allocation concealment, blinding of participants and personnel, blinding of outcome assessment, incomplete outcome data, selective reporting, and other biases.

### 2.4. Statistical analyses

The meta-analysis was conducted using the Nordic Cochrane Center Review Manager (version 5.4). Incisional hernia and SSI were binary variables, and odds ratios (ORs) with 95% confidence intervals (CIs) were used. For continuous variables, operating time, hospital stay, incisional hernia, and wound healing time were calculated as mean differences (MDs) with a 95% CI. For the heterogeneity tests, we used the χ^2^ and *I*^2^ inconsistency statistics. The values of the statistics above 50% were considered significant, and the possible reasons were explored in greater detail. Fixed effect models were adopted because of low heterogeneity among the studies. If the heterogeneity among the studies was high, the reasons were further analyzed, and a random-effects model was adopted. Publication bias was evaluated using funnel plots.

## 3. Results

### 3.1. Study characteristics

**Figure 1.**
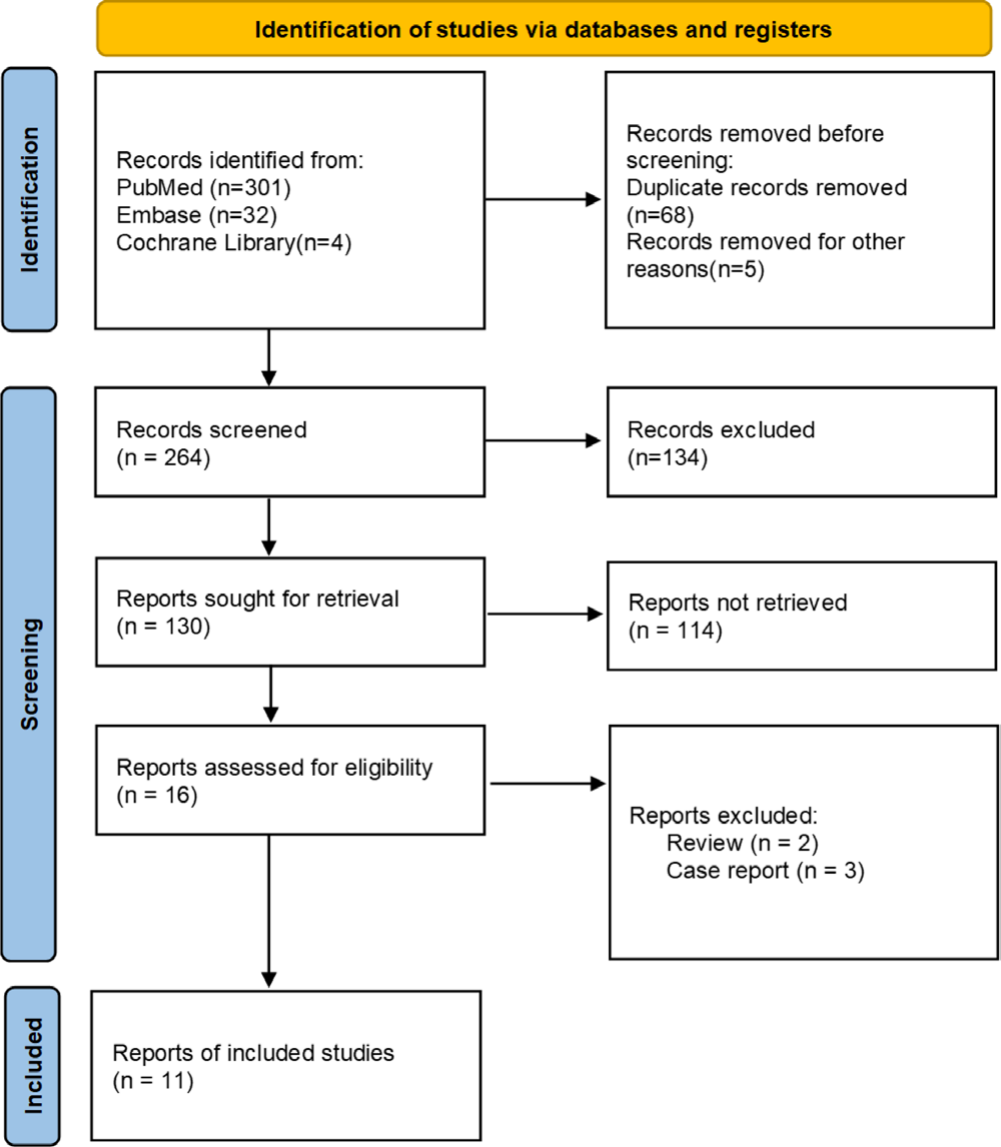
Flowchart of the search strategy.

**Figure 2.**
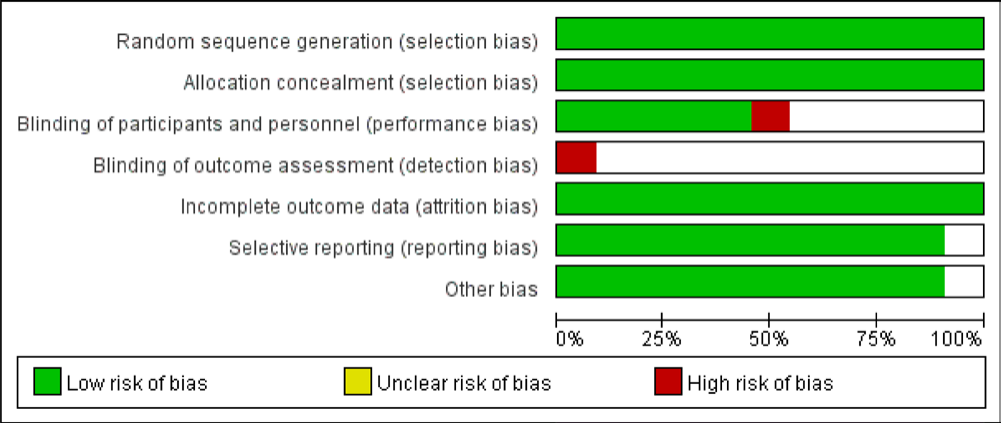
Summary of risk of bias assessment.

From the initial search of databases and other sources, 326 studies were identified. A flowchart was used to identify the eligible studies (Fig. 1). Eleven articles were selected, and 995 patients (506 patients in the PSC group and 489 patients in the LC group) were included. Detailed information regarding the basic characteristics of the study is presented in Table 1. An assessment of the bias is presented in Figure 2.

**Table 1:** Characteristics of the studies included in the meta-analysis.

First author	Country, year	Patients (PSC/LC)	Stoma type	Prophylactic antibiotic use	Postoperative antibiotic use	Follow-up (mo)	SSI (PSC/LC)	Incision hernia (PSC/LC)	Operative time (min) (PSC/LC)	hospital stay (d) (PSC/LC)	Wound healing time(wk) (PSC/LC)
Ali^[10]^	Pakistan, 2021	37/35	Ileostomy	NM	NM	3	2/8	0/1	142.3 ± 7.04/150 ± 9.85	5 ± 1.33/7 ± 1.17	NM
Alvandipour^[11]^	Iran, 2016	34/32	Ileostomy	NM	NM	3	1/7	NM	71.76 ± 9/69.84 ± 10	6.55 ± 1.18/6.78 ± 1.53	NM
Amano^[12]^	Japan, 2018	80/79	Ileostomy, colostomy	1 g cefmetazole/2-generation cephalosporins	A single additional dose was given 1 h after completion of the surgery	1	4/7	NM	NM	NM	NM
Camacho-Mauries^[13]^	Mexico, 2013	31/30	Ileostomy, colostomy	1 g amoxicillin, clavulanate	NM	12	0/11	0/2	123.5 (30–300)/131 (40–330)	8.4/7.2	3.8/5.9
Carannante^[14]^	Italy, 2024	58/59	Ileostomy	Second-generation cephalosporins or fuoroquinolone and metronidazole in case of penicillin allergy	No	6	3/11	NM	NM	3.86 ± 0.28/4.13 ± 0.23	NM
Dusch^[15]^	Germany, 2013	43/41	Ileostomy	Single dose of antibiotic	NM	6	0/10	5/1	84.1 ± 29.9/94.8 ± 26.6	6.0 ± 4.1/6.0 ± 3.4	NM
Lee^[16]^	South Korea, 2014	58/55	Ileostomy, colostomy	2-generation cephalosporins or fluoroquinolone and metronidazole	No	1	1/8	NM	111 (65)/122 (73)	9.1 (7)/9.7 (8.5)	35/24
Lopez^[17]^	Philippines, 2015	61/60	Ileostomy, colostomy	Intravenous antibiotics	No	1	1/6	NA	NA	NA	NA
O′Leary^[18]^	Ireland, 2017	34/27	Ileostomy	1.2 g augmentin	NM	6	3/8	NA	71 ± 38.3/63.1 ± 15.1	7.9 ± 6.3/8.4 ± 5.8	NA
Reid^[19]^	Australia, 2010	30/31	Ileostomy	1 g cefazolin/500 mg metronidazole	NM	12	2/12	NA	48.4 (19.0)/48.2 (22.0)	5.5 (3.9)/6.1 (3.5)	20.6 (11.9)/24.6 (10.4)
Sureshkumar^[20]^	India, 2018	40/40	Ileostomy, colostomy	Yes	NM	3	3/17	0/4	142.13/149.5	9.9/11.95	NM

LC = linear closure, NA = no data, NM = not mention, PSC = purse-string closure, SSI = surgical site infection.

### 3.2. Surgical site infection

**Figure 3.**
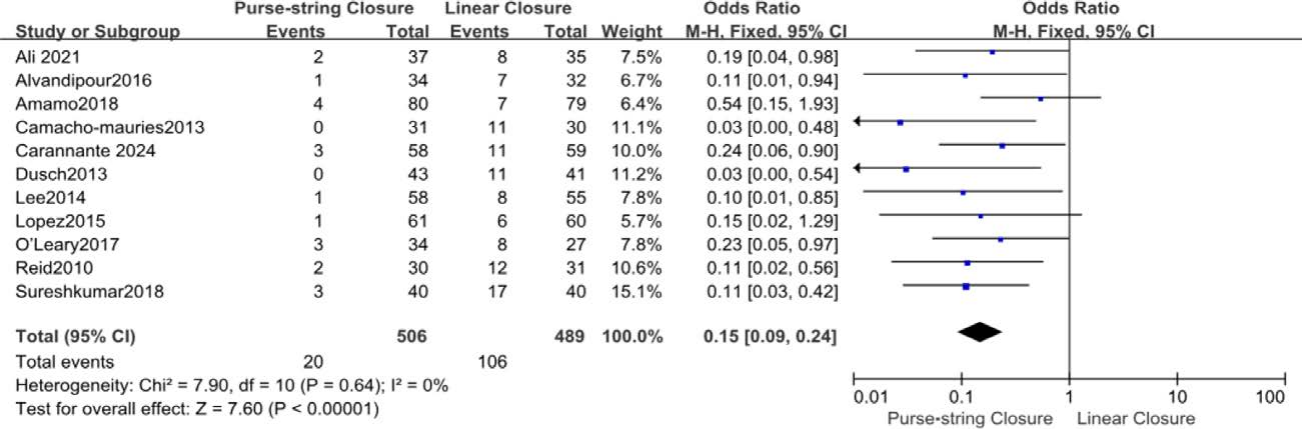
Forest plot of SSI. CI = confidence interval, SSI = surgical site infection, df = degrees of freedom, M-H = Mantel-Haenszel.

A total of 995 patients in 11 trials reported SSI.^[10–20]^ There was a significant reduction in SSI in patients who underwent PSC (OR, 0.15; 95% CI, 0.09–0.24; *P* < .00001). No significant heterogeneity was observed among the study results (*I*² = 0%). A fixed-effects model was employed to validate the findings (Fig. 3).

### 3.3. Incisional hernia

**Figure 4.**
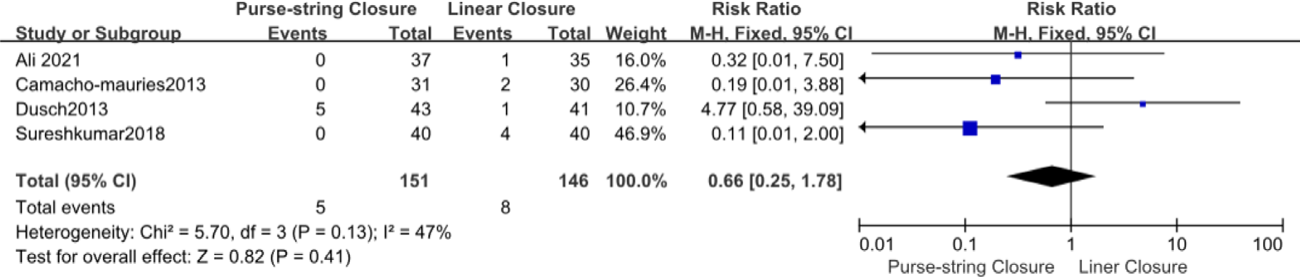
Forest plot of incisional hernia. CI = confidence interval, df = degrees of freedom, M-H = Mantel-Haenszel.

A total of 297 patients in 4 studies reported incisional hernia.^[10,13,15,20]^ Compared to the LC group (5.48%), the PSC group had a lower rate of incisional hernias (3.31%). However, the difference was not statistically significant (OR, 0.66; 95% CI, 0.25–1.78; *P *= .41). A low significant heterogeneity was reported among the study results (*I*^2^ = 47%). A fixed-effects model was used to validate the findings (Fig. 4).

### 3.4. Operation time

**Figure 5.**
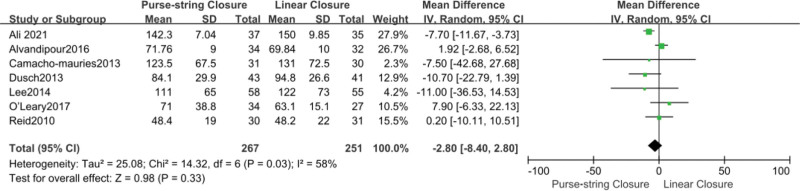
Forest plot of operative time. CI = confidence interval, SD = standard deviation, df = degrees of freedom, IV = Iterative Moment Method.

Six studies with a total of 518 participants reported the operation time. The differences between the 2 groups were not statistically significant (MD, −2.80; 95% CI, −8.40 to 2.80; *P* = .33). Owing to the high heterogeneity (*I*^2^ = 58%), we used a random-effects model to validate the results (Fig. 5).

### 3.5. Length of hospital stay

**Figure 6.**
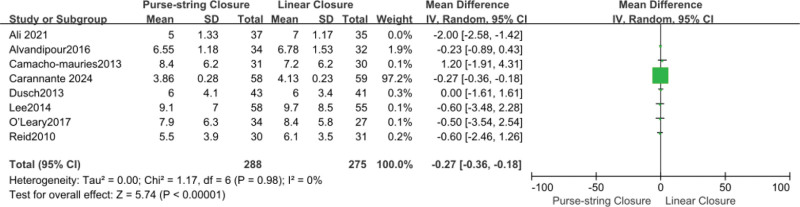
Forest plot of hospital stay. CI = confidence interval, SD = standard deviation, df = degrees of freedom, IV = Iterative Moment Method.

Six articles reported on the length of hospital stay, including 446 patients. The pooled data did not reveal any significant differences (MD, −0.58; 95% CI, −1.33 to 0.16; *P* = .12). No significant heterogeneity was observed among the study results (*I*² = 80%). The heterogeneity decreased after the study by Ali et al^[10]^ was excluded, and there were still no statistical differences between these 2 groups (MD, −0.27; 95% CI, −0.36 to −0.18; *P* < .00001; *I*² = 0%) (Fig. 6).

### 3.6. Wound healing time

**Figure 7.**
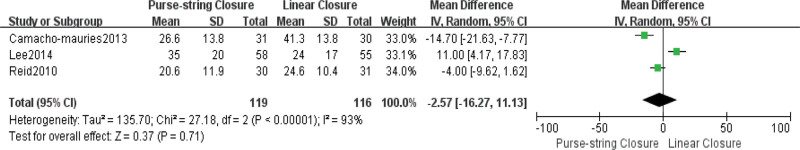
Forest plot of wound healing time. CI = confidence interval, SD = standard deviation, df = degrees of freedom, IV = Iterative Moment Method.

The healing time of the wound was reported in 3 studies with 235 participants. There was no statistically significant difference (MD, −2.57; 95% CI, −16.27 to 11.13; *P* = .71; *I*^2^ = 93%). We used the random-effects methodology (Fig. 7) to validate the results because of the statistical significance of the heterogeneity (*P* < .00001).

### 3.7. Publication bias

**Figure 8.**
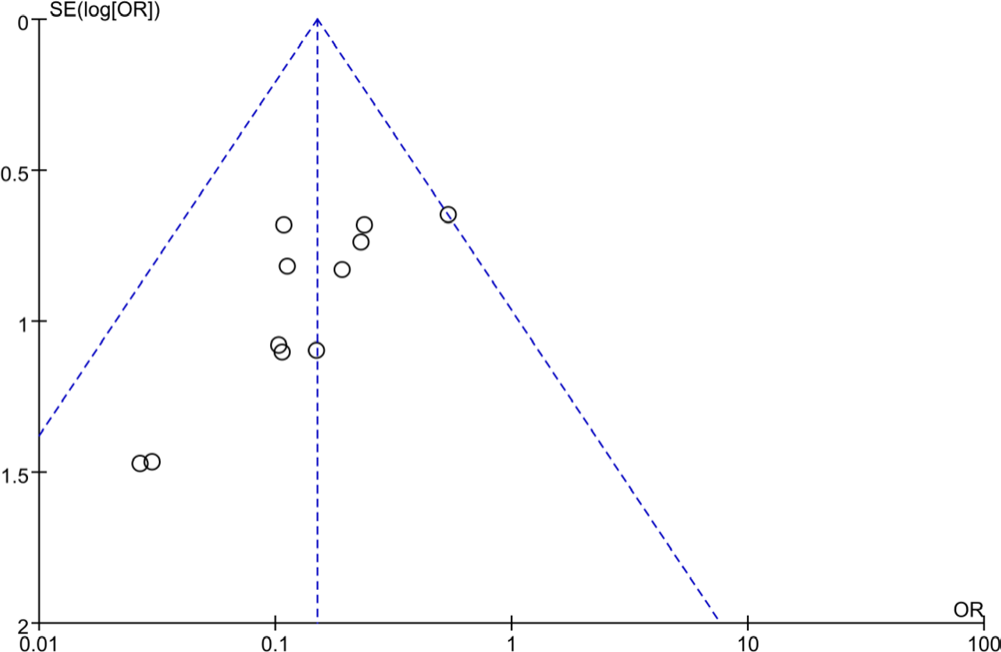
Funnel plot of SSI. OR = odds ratio, SE = standard error, SSI = surgical site infection.

The 11 studies included in this meta-analysis are symmetrically distributed around the vertical dotted line that indicates the overall effect estimate. Consequently, the funnel plot (Fig. 8) does not reveal any evidence of publication bias or other forms of reporting bias.

## 4. Discussion

We included 11 recently published RCTs that compared the surgical outcomes of PSC and LC approaches following stoma reversal surgery. This meta-analysis indicated that the PSC technique was associated with a significantly lower SSI rate than the LC technique without increasing operation time and hospital stays.

Several methods have been described that may reduce the risk of SSI.^[21–23]^ The meta-analysis by Li et al^[24]^showed that PSC is the best skin closure technique following stoma reversal in terms of SSI rat compared with wound closure with a drain, secondary closure, loose primary closure, and delayed primary closure. The results are consistent with our analysis. Analysis of data from 11 RCTs including a total of 995 participants showed that PSC likely reduces the risk of SSI in patients undergoing stoma reversal. Stoma reversal is associated with a high risk of SSI owing to the presence of microorganisms on the skin around the stoma site and possible contamination with the intestinal content during bowel manipulation.^[25,26]^ Because superficial skin heals more quickly, bacterial contamination is not easily discharged following linear suture treatment, leading to local abscess formation.^[27]^ In the purse-string procedure after suturing the skin, a skin defect (0.5–1 cm) is left or centrally opened. It prevents the accumulation of exudate and maintains the persistent drainage of fluid at an easily contaminated stoma reversal wound.^[28]^ The wound healed secondarily with small granulation tissue and achieved a better cosmetic outcome. Meanwhile, the heterogeneity among the studies was low. Therefore, we believe that PSC offers distinct advantages for stoma reversal in real practice.

Our study did not reveal a statistically significant difference in the incidence of incisional hernia, length of hospital stays, or wound healing time between the 2 surgical procedures. For most patients, the SSI was superficial after stoma reversal and did not increase the damage to the strength of the abdominal wall structure. That won’t increase the incidence of incisional hernia. This may explain why the incidence of incisional hernia is statistically equivalent between the 2 groups.

Due to the heterogeneity among the available studies,^[27,29]^ more RCTs with a large sample size, multicenter design, and long follow-up are needed.

## 5. Conclusion

In conclusion, our meta-analysis showed that skin closure following stoma reversal using the PSC technique was more advantageous than the LC technique.

## Acknowledgments

We thank the support from Zigong Fourth People’s Hospital and funding support from the Health Commission of Zigong City (21yb046).

## Author contributions

**Formal analysis:** Jinlong Luo, Dan Liu, Junmei Wu, Jin Chen, Lie Yang.

**Writing – original draft:** Jinlong Luo, Huaiwu Jiang.

**Writing – review & editing:** Jinlong Luo, Hua Yang, Lie Yang.

## References

[1] Lindner S Eitelbuss S Hetjens S. Less is more-the best test for anastomotic leaks in rectal cancer patients prior to ileostomy reversal. Int J Colorectal Dis. 2021;36:2387–98. 34251505 10.1007/s00384-021-03963-1 PMC8505329

[2] Seidelman J Anderson DJ. Surgical site infections. Infect Dis Clin North Am. 2021;35:901–29. 34752225 10.1016/j.idc.2021.07.006

[3] Gachabayov M Lee H Chudner A Dyatlov A Zhang N Bergamaschi R. Purse-string vs. linear skin closure at loop ileostomy reversal: a systematic review and meta-analysis. Tech Coloproctol. 2019;23:207–20. 30809775 10.1007/s10151-019-01952-9

[4] Sun Z Zhao Y Liu L Qin J. Clinical outcomes of ileostomy closure before adjuvant chemotherapy after rectal cancer surgery: an observational study from a chinese center. Gastroenterol Res Pract. 2021;2021:5592721. 34335738 10.1155/2021/5592721 PMC8294951

[5] Banerjee A. Pursestring skin closure after stoma reversal. Dis Colon Rectum. 1997;40:993–4. 9269819 10.1007/BF02051210

[6] Page MJ Mckenzie JE Bossuyt PM Boutron I Moher D. The prisma 2020 statement: an updated guideline for reporting systematic reviews. PLoS Med. 2021;18:e1003583. 33780438 10.1371/journal.pmed.1003583 PMC8007028

[7] Stroup DF Berlin JA Morton SC. Meta-analysis of observational studies in epidemiology: a proposal for reporting. Meta-Analysis of Observational Studies in Epidemiology (MOOSE) group. JAMA. 2000;283:2008–12. 10789670 10.1001/jama.283.15.2008

[8] Mangram AJ Horan TC Pearson ML Silver LC Jarvis WR. Guideline for Prevention of Surgical Site Infection, 1999. Centers for Disease Control and Prevention (CDC) Hospital Infection Control Practices Advisory Committee. Am J Infect Control. 1999;27:97–132; quiz 133. 10196487

[9] Qumseya BJ. Quality assessment for systematic reviews and meta-analyses of cohort studies. Gastrointest Endosc. 2021;93:486–94.e1. 33068610 10.1016/j.gie.2020.10.007

[10] Ali D Zubair M Kaiser MA Khokhar I Afzal MF. Outcome of purse-string versus linear skin closure after ileostomy stoma reversal in terms of stoma sites infection and cosmesis. J Pak Med Assoc. 2021;71:414–6. 33819218 10.47391/JPMA.05-673

[11] Alvandipour M Gharedaghi B Khodabakhsh H Karami MY. Purse-string versus linear conventional skin wound closure of an ileostomy: a randomized clinical trial. Ann Coloproctol. 2016;32:144–9. 27626025 10.3393/ac.2016.32.4.144 PMC5019967

[12] Amano K Ishida H Kumamoto K. Correction to: purse-string approximation vs. primary closure with a drain for stoma reversal surgery: results of a randomized clinical trial. Surg Today. 2019;49:231–7. 30367238 10.1007/s00595-018-1729-5

[13] Camacho-Mauries D Rodriguez-Díaz J Salgado-Nesme N González Q Vergara-Fernández O. Randomized clinical trial of intestinal ostomy takedown comparing pursestring wound closure vs conventional closure to eliminate the risk of wound infection. Dis Colon Rectum. 2013;56:205–11. 23303149 10.1097/DCR.0b013e31827888f6

[14] Carannante F Costa G Miacci V. Comparison of purse-string technique vs linear suture for skin closure after ileostomy reversal. A randomized controlled trial. Langenbecks Arch Surg. 2024;409:141. 38676785 10.1007/s00423-024-03332-w

[15] Dusch N Goranova D Herrle F Niedergethmann M Kienle P. Randomized controlled trial: comparison of two surgical techniques for closing the wound following ileostomy closure: purse string vs direct suture. Colorectal Dis. 2013;15:1033–40. 23634717 10.1111/codi.12211

[16] Lee JT Marquez TT Clerc D Gie O Christoforidis D. Pursestring closure of the stoma site leads to fewer wound infections: results from a multicenter randomized controlled trial. Diseases of the Colon Rectum. 2014;57:1282–9. 25285695 10.1097/DCR.0000000000000209

[17] Lopez MP Melendres MFA Maglangit SACA Roxas MFT Monroy HJ Crisostomo AC. A randomized controlled clinical trial comparing the outcomes of circumferential subcuticular wound approximation (CSWA) with conventional wound closure after stoma reversal. Tech Coloproctol. 2015;19:461–8. 26045008 10.1007/s10151-015-1322-5

[18] O’Leary DP Carter M Wijewardene D. The effect of purse-string approximation versus linear approximation of ileostomy reversal wounds on morbidity rates and patient satisfaction: the “STOMA” trial. Tech Coloproctol. 2017;21:863–8. 29149428 10.1007/s10151-017-1713-x

[19] Reid K Pockney P Pollitt T Draganic B Smith SR. Randomized clinical trial of short-term outcomes following purse-string versus conventional closure of ileostomy wounds. Br J Surg. 2011;97:1511–7. 10.1002/bjs.7151 20575111

[20] Sureshkumar S Jubel K Ali MS. Comparing surgical site infection and scar cosmesis between conventional linear skin closure versus purse-string skin closure in stoma reversal - a randomized controlled trial. Cureus. 2018;10:e2181. 29657907 10.7759/cureus.2181 PMC5896871

[21] Lin H Li WZ. Complete closure using a double purse-string closure for skin defects. Dermatol Surg. 2010;35:1406–9. 10.1111/j.1524-4725.2009.01248.x 19549181

[22] Lim JT Shedda SM Hayes IP. Gunsight” skin incision and closure technique for stoma reversal. Dis Colon Rectum. 2010;53:1569–75. 20940608 10.1007/DCR.0b013e3181f0535a

[23] Li CK Liang WW Wang HM. Gunsight sutures significantly reduce surgical-site infection after ileostomy reversal compared with linear sutures. Gastroenterol Rep (Oxf). 2020;9:357–62. 34567568 10.1093/gastro/goaa075 PMC8460110

[24] Li LT Hicks SC Davila JA. Circular closure is associated with the lowest rate of surgical site infection following stoma reversal: a systematic review and multiple treatment meta-analysis. Colorectal Dis. 2014;16:406–16. 24422861 10.1111/codi.12556

[25] Kobayashi S Ito M Sugito M Kobayashi A Nishizawa Y Saito N. Association between incisional surgical site infection and the type of skin closure after stoma closure. Surg Today. 2011;41:941–5. 21748610 10.1007/s00595-010-4405-y

[26] Liang MK Li LT Avellaneda A Moffett JM Hicks SC Awad SS. Outcomes and predictors of incisional surgical site infection in stoma reversal. Jama Surg. 2013;148:183–9. 23426597 10.1001/jamasurgery.2013.411

[27] Sang IY Sun MB Namgung H Dong GP. clinical trial on the incidence of wound infection and patient satisfaction after stoma closure: comparison of two skin closure techniques. Ann Coloproctol. 2015;31:29–33. 25745624 10.3393/ac.2015.31.1.29 PMC4349913

[28] Gulzar MR Aslam F Farooq MU Ahmad S Adil S Tahir S. Comparative study of linear closure technique versur purse string closure technique of skin closure in stoma reversal. Professional Med J. 2020;27:1038–42.

[29] Bhangu A Nepogodiev D Futaba K; West Midlands Research Collaborative. Systematic review and meta-analysis of the incidence of incisional hernia at the site of stoma closure. World J Surg. 2012;36:973–83. 22362042 10.1007/s00268-012-1474-7

